# Effect of oseltamivir phosphate versus placebo on platelet recovery and plasma leakage in adults with dengue and thrombocytopenia; a phase 2, multicenter, double-blind, randomized trial

**DOI:** 10.1371/journal.pntd.0010051

**Published:** 2022-01-07

**Authors:** Rahajeng N. Tunjungputri, Silvita Fitri Riswari, Setyo G. Pramudo, Lydia Kuntjoro, Bachti Alisjahbana, Harry Galuh Nugraha, Andre van der Ven, Muhammad Hussein Gasem, Quirijn de Mast

**Affiliations:** 1 Department of Internal Medicine and the Radboud Center for Infectious Diseases, Radboud University Medical Center, Nijmegen, The Netherlands; 2 Center for Tropical and Infectious Disease (CENTRID), Faculty of Medicine Diponegoro University, Dr. Kariadi Hospital, Semarang, Indonesia; 3 Research Center for Care and Control of Infectious Disease (RC3ID), Universitas Padjadjaran, Bandung, Indonesia; 4 Parasitology Division, Department of Biomedical Sciences, Faculty of Medicine, Universitas Padjadjaran, Bandung, West Java, Indonesia, Indonesia; 5 Department of Internal Medicine, Diponegoro National University Hospital, Faculty of Medicine Diponegoro University, Semarang, Central Java, Indonesia; 6 Department of Radiology, Diponegoro National University Hospital, Faculty of Medicine Diponegoro University, Semarang, Central Java, Indonesia; 7 Department of Internal Medicine, Hasan Sadikin General Hospital, Faculty of Medicine Universitas Padjadjaran, Bandung, West Java, Indonesia, Indonesia; 8 Department of Radiology, Hasan Sadikin General Hospital, Faculty of Medicine Universitas Padjadjaran, Bandung, West Java, Indonesia, Indonesia; CDC, UNITED STATES

## Abstract

**Background:**

Thrombocytopenia, bleeding and plasma leakage are major complications of dengue. Activation of endogenous sialidases with desialylation of platelets and endothelial cells may underlie these complications. We aimed to assess the effects of the neuraminidase inhibitor oseltamivir on platelet recovery and plasma leakage in dengue.

**Methods:**

We performed a phase 2, double-blind, multicenter, randomized trial in adult dengue patients with thrombocytopenia (<70,000/μl) and a duration of illness ≤ 6 days. Oseltamivir phosphate 75mg BID or placebo were given for a maximum of five days. Primary outcomes were the time to platelet recovery (≥ 100,000/μl) or discharge from hospital and the course of measures of plasma leakage.

**Results:**

A total of 70 patients were enrolled; the primary outcome could be assessed in 64 patients (31 oseltamivir; 33 placebo). Time to platelet count ≥100,000/μl (n = 55) or discharge (n = 9) were similar in the oseltamivir and placebo group (3.0 days [95% confidence interval, 2.7 to 3.3] vs. 2.9 days [2.5 to 3.3], *P* = 0.055). The kinetics of platelet count and parameters of plasma leakage (gall bladder thickness, hematocrit, plasma albumin, syndecan-1) were also similar between the groups.

**Discussion:**

In this trial, adjunctive therapy with oseltamivir phosphate had no effect on platelet recovery or plasma leakage parameters.

**Trial registration:**

ISRCTN35227717.

## Author summary

Moderate to severe thrombocytopenia is common in the febrile and/or critical phase of dengue virus infection. Platelets are important for preservation of vascular integrity, especially during inflammation, and low platelet counts may contribute to plasma leakage. Currently, no therapeutic intervention that targets the pathogenic pathway is available for DENV infection, including therapies to prevent or reduce thrombocytopenia or plasma leakage. Oseltamivir phosphate is widely used for prevention and treatment of influenza by inhibiting viral neuraminidase. However, oseltamivir may also inhibit human endogenous neuraminidase involved in sialic acid metabolism, and as such extend the lifespan of platelets. In the phase 2 TOTO trial (**T**reatment **O**f **T**hrombocytopenia with **O**seltamivir in acute dengue virus infection: a randomized, placebo controlled, multicenter trial) we investigated the potential of oseltamivir phosphate to shorten the time to platelet recovery and reduce plasma leakage in patients with DENV infection. In this trial involving 70 adult thrombocytopenic patients, hospitalized with acute DENV infection, adjunctive therapy with oseltamivir phosphate did not shorten platelet recovery time compared with placebo. The trial also did not show an effect of adjunctive oseltamivir on plasma leakage parameters. The reasons that oseltamivir had no apparent effect on platelet counts, markers of plasma leakage and glycocalyx distortion in this study remain speculative, but may involve one or more of the following; first, dengue-associated thrombocytopenia and plasma leakage are both multifactorial in origin and targeting neuraminidase alone may be insufficient to impact these processes. Second, oseltamivir phosphate was designed to inhibit viral neuraminidase, and data of its inhibitory actions on human neuraminidases are inconclusive. The finding in this study also suggest that while laboratory works may lead to hypotheses for novel treatment, proof of concept studies are essential to test them in a clinical setting.

## Background

Dengue is the most common arboviral infection globally and is associated with a substantial global economic burden [1]. Dengue virus (DENV) infection may be asymptomatic or result in clinical manifestations ranging from a mild febrile illness to a life-threatening shock syndrome. The latter usually occurs around or shortly after the time of defervescence during the so-called critical phase. Endothelial dysfunction leading to a transient vascular leak syndrome and bleeding are hallmarks of severe dengue. Moderate to severe thrombocytopenia is common in the febrile and/or critical phase of dengue and severe manifestations of dengue are usually preceded by a rapid drop in platelet count [2]. Different guidelines advise to measure platelet count daily and it is a commonly used parameter to guide timing of hospital admission and discharge [3].

Thrombocytopenia and platelet dysfunction may contribute to bleeding manifestations in dengue [4–7], which usually manifest as skin or mucosal bleeding and can be life-threatening. In addition, an increasing body of evidence highlights a role for platelets in maintaining vascular homeostasis, both in inflammatory conditions, as well as in absence of injury or inflammation [8–10]. Nontheless, it should be acknowleged that thrombocytopenia is also common in patients with non-severe dengue and that platelets may release pathogenic molecules. This highlights the complex and still incompletely understood role of platelets in dengue pathophysiology.

Currently, no therapeutic intervention that targets the virus or pathogenic pathways is available for DENV infection, including therapies to prevent or reduce thrombocytopenia or plasma leakage. Intravenous immunoglobulins or corticosteroids were ineffective in treating thrombocytopenia [11,12], whereas a large multicenter trial failed to demonstrate a beneficial effect of prophylactic platelet transfusion [13].

The cause of thrombocytopenia in dengue is multifactorial, including increased clearance and reduced production [14]. Both platelets and endothelial cells have an abundance of sialic acids on their surface. Loss of sialic acid residues from platelet glycoproteins by endogenous sialidases, such as neuraminidase-1 and neuraminidase-3, results in rapid removal of platelets from the circulation by the hepatic Aswell Morell receptor [15]. This is a physiological clearance mechanism of senescent platelets, but may also lead to accelerated platelet clearance in pathological conditions. We recently showed that platelet desialylation also occurs in dengue [16]. In addition, DENV non-structural protein-1 (NS1) activates sialidases in endothelial cells leading to desialylation and disruption of the endothelial glycocalyx and vascular leak [17,18]. Oseltamivir phosphate is widely used for prevention and treatment of influenza by inhibiting viral neuraminidase. However, oseltamivir may also inhibit human endogenous neuraminidase involved in sialic acid metabolism [19], and as such extend the lifespan of platelets. Studies indeed showed a significant increase in platelet number in individuals prescribed oseltamivir for (suspected) influenza [20,21], and the successful use of oseltamivir to increase platelet numbers in immune thrombocytopenia (ITP) [22–25] and sepsis [26].

In the phase 2 TOTO trial (**T**reatment **O**f **T**hrombocytopenia with **O**seltamivir in acute dengue virus infection: a randomized, placebo controlled, multicenter trial) we investigated the potential of oseltamivir phosphate to shorten the time to platelet recovery and reduce plasma leakage in patients with DENV infection.

## Methods

### Ethics statement

The study protocol was approved by the ethical review boards of Faculty of Medicine Diponegoro University and Universitas Padjajaran as well as the Indonesian National Agency of Drug and Food Control. All participants provided written informed consent.

### Study population and sample size calculation

Patients were recruited among patients hospitalized for suspected DENV infection in six hospitals in Central and West Java Indonesia: RS Nasional Diponegoro, RSUD K.R.M.T. Wongsonegoro, William Booth Hospital, RSUD Kartini, Hasan Sadikin General Hospital and RSAU dr. M. Salamun; recruitment in the two latter hospitals was started halfway the trial to increase patient recruitment. Patients were eligible for inclusion if they were aged at least 16 years; had a platelet count below 70,000/μl; had fever for six days or less; were positive for DENV NS-1 or positive for acute dengue serology with probable dengue criteria defined in WHO 2009. Restriction of enrolment to participants aged 16 years and above was done for safety and ethical reasons and because we considered it unlikely that fundamental differences exist in the pathophysiology of dengue-associated thrombocytopenia and plasma leakage between adults and children.

Patients were excluded when they used platelet function inhibitors or anticoagulants; had an estimated creatinine clearance <70 ml/min and/or an alanine aminotransferase (ALT) value >3x ULN; were pregnant or breastfeeding; had a platelet transfusion during the current hospitalization; had an already recovering platelet number; or had persistent or recurrent clinically significant bleeding. All patients provided written informed consent.

### Study design and procedures

This was a phase 2, double-blind, multicenter, randomized, placebo-controlled trial.

Eligible patients were randomized using block randomization in a 1:1 ratio to receive oral oseltamivir phosphate 75mg twice daily or placebo. Trial drugs were administered orally under supervision until the primary endpoint was reached or until a maximum of five days of treatment. In patients with an estimated creatinine clearance between 30 and 60 ml/min, a dose reduction of 75mg OD was used. Generic oseltamivir phosphate was manufactured by PT Indofarma (Bekasi, Indonesia) in compliance with international manufacturing practice standards; trial drugs were prepared by Kimia Farma (Jakarta, Indonesia).

Patients were followed daily by members of the study team for up to five days of treatment or until hospital discharge. Daily assessments included assessment of possible side effects and bleeding manifestations (WHO bleeding score), ultrasonography (Philips Lumify portable ultrasound) to assess the thickness of the gall bladder wall and the presence of ascites and pleural fluid, and laboratory examinations. The latter consisted of a complete blood count (Hb, hematocrit, leukocytes, platelet count) twice daily using a standard hematology analyzer and daily plasma creatinine and ALT concentration. Plasma concentrations of albumin (ALB Flex Dimension, Siemens Healthcare Diagnostics, Ltd) and syndecan-1 (human syndecan-1 ELISA kit, Abcam, ab46506) were determined in stored plasma. Patients who were discharged before their platelet numbers had reached 100,000/μl were visited at home whenever possible. A post-discharge visit was scheduled approximately three weeks after enrolment to assess for possible late complications and to obtain reconvalescence laboratory measurements.

An independent data monitor and an independent safety monitor reviewed study data at predefined intervals. On-site monitoring visits were performed by the data monitor and the Indonesian Food and Drug Authority (*BPOM*, *Badan Pengawas Obat dan Makanan*).

### Outcomes

Primary outcomes measures were: (a) the time to platelet recovery, defined as the time between study enrolment and the platelet count reaching a value of ≥ 100,000/μl, or discharge from hospital when the platelet number was still <100,000/μl and without follow-up samples at home, and (b) measures of plasma leakage, including hematocrit, concentrations of plasma albumin and the glycocalyx marker syndecan-1 and results of daily ultrasonography (gall bladder wall thickness; presence of ascites or pleural fluid). Secondary outcome parameters included safety, the rate of change of platelet count at 24 and 48 hours and 5 days, occurrence of severe thrombocytopenia and the occurrence of clinical bleeding.

### Statistical analyses

The sample size calculation was based on data from a previous study in dengue patients in the same area [16]. We assumed that a mean time for platelet number to reach 100,000/μl in those with enrolment platelet count of less than 70,000/μl was 4 days (SD 1.4). Considering a shortening of platelet recovery to 3 days in the oseltamivir group as clinically relevant, 31 participants per group had to be enrolled using a two-sided approach (alpha 0.05) with power 80%. Accounting loss to follow up, enrolment of 35 participants per group was planned.

All efficacy analyses were conducted in the intention-to-treat population (which included all patients who underwent randomization). The time to platelet count recovery or discharge was compared with the use of a two-sided Student T-test. Differences between the groups in kinetics of daily platelet count, hematocrit, plasma albumin and syndecan-1 concentrations and gallbladder wall thickness were compared using mixed model methods. Differences in the percentage of participants with pleural fluid or ascites were analyzed using Chi square test. All analyses were performed using Graphpad Prism 9 (Graphpad).

## Results

A total of 70 patients were randomly assigned to receive oseltamivir (35 patients) or placebo (35 patients) during the period from January 2018 to February 2019. Demographic and baseline disease characteristics were generally similar in the two groups, except for ascites being more common in the placebo group at baseline (**Table 1**).

**Table 1:** Characteristics of study participants.

	Oseltamivir	Placebo	*P* value
Number (%)	35	35	
Male sex, n (%)	18 (51)	25 (71)	0.09
Age, years	27.5 (9.8)	27.6 (11.3)	0.95
Days of illness, days	4.4 (1.1)	4.7 (0.9)	0.19
NS1 positive, n (%)	25 (71.4)	21 (60)	0.31
Bleeding events, n (%)			
Epistaxis	5 (14.2)	3 (8.6)	0.46
Skin petechiae	10 (28.2)	12 (34.3)	0.60
Melena	1 (2.8)	1 (2.8)	0.99
Hematuria	2 (5.7)	0 (0)	0.31
Hemoglobin, g/dl	14.7 (1.8)	14.7 (1.8)	0,98
Hematocrit, %	42.8 (4.9)	43.4 (5.7)	0.65
Leukocytes, x10^3^/μl	4.3 (2.4)	4.3 (2.2)	0.50
Platelets, cells/μl	44,000 (23,000)	43,000 (16,000)	0.10
Ureum, mg/dl	15.7 (3.9)	23.4 (5.1)	0.15
Creatinine, mg/dl	0.9 (0.3)	0.8 (0.2)	0.18
ALT, IU/l	57.2 (38.5)	71.4 (50.3)	0.19
Albumin, g/dl	2.76 (0.3)	2.86 (0.4)	0.39
Ultrasonography			
Gallbladder thickness, mm	3.63 (1.47)	3.84 (1.59)	0.56
Pleural effusion, n (%)	4 (11.4)	2 (5.7)	0.41
Ascites, n (%)	3 (8.6)	10 (28.5)	0.03

Data are mean and standard deviation unless otherwise indicated. *P* values represent difference between oseltamivir and placebo group (Mann-Whitney U-test or chi-square test when appropriate).

The mean duration of illness at enrolment was 4.4 days (1.1 days) in the oseltamivir group and 4.7 days (0.9 days) in the placebo group (P = 0.19). Randomization and receipt of intervention was done immediately following enrolment. The platelet count at enrolment was similar in both groups with a mean (SD) value of 44,000/μl (23,000/μl) in the oseltamivir group and 43,000/μl (16,000/μl) in the placebo group. Dengue was diagnosed by a positive NS1 rapid test in 46 (66%) patients and by serology in the remainder; serotyping by PCR was not perfomed. Twenty nine of all 70 patients had positive IgG, suggestive of a secondary infection in these patients. According to the 2009 WHO Guideline, none of the patients classified as severe dengue.

**Fig 1:**
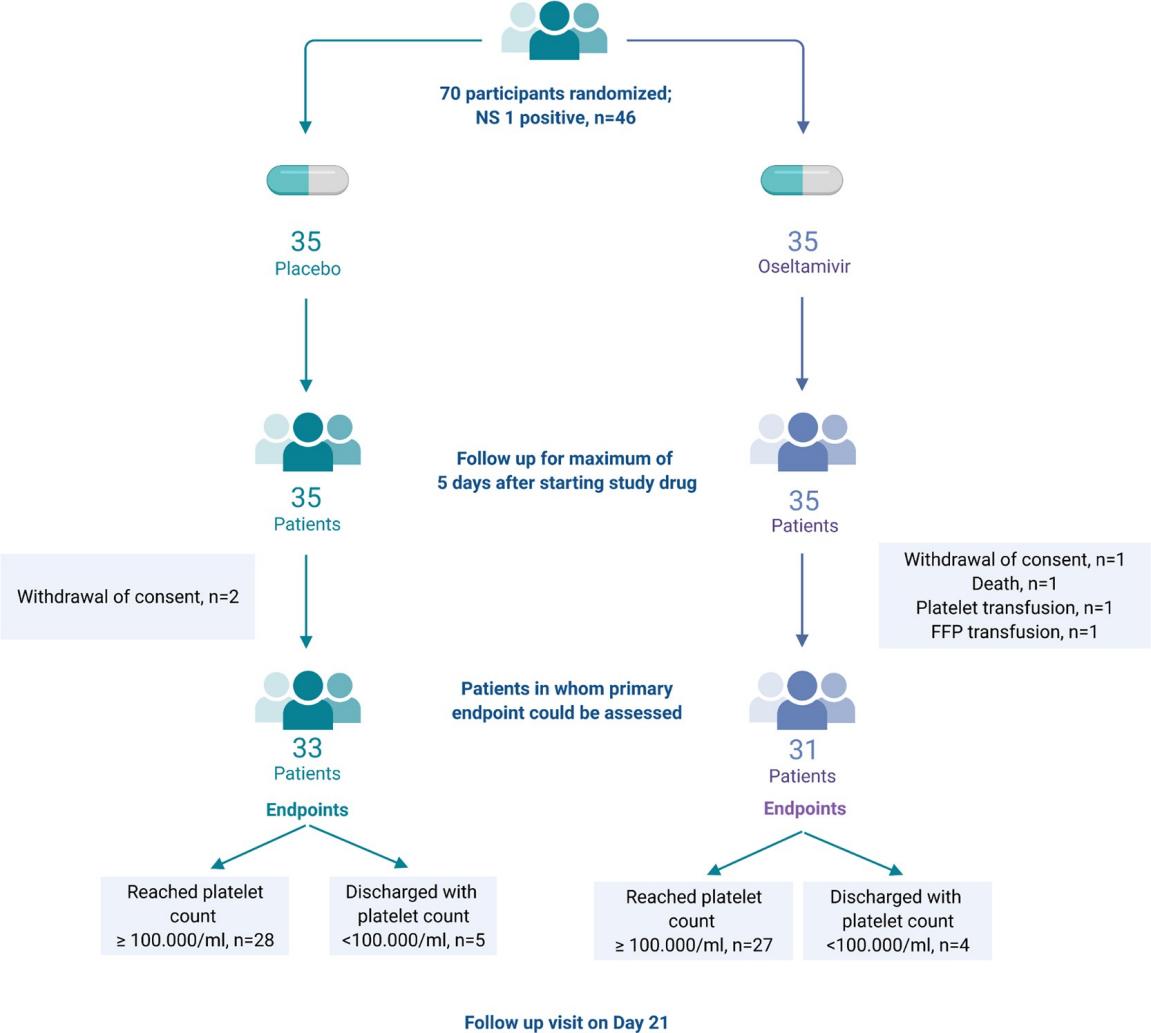
Study flow chart.

**Fig 1** shows the trial profile. All the participants received at least one dose of the assigned oseltamivir or placebo. The trial regimen was prematurely discontinued in five different patients, because of withdrawal of consent (one in osseltamivir group and two in placebo group), administration of a platelet transfusion (one in oseltamivir group) or fresh frozen plasma (FFP) transfusion (one in oseltamivir group). One patient assigned to the oseltamivir group died. Seven patients in the oseltamivir group and three in the placebo group received less than 4 doses of the study drug; an overview of the number of study medication doses taken is given in S1 Fig.

### Platelet recovery

**Fig 2:**
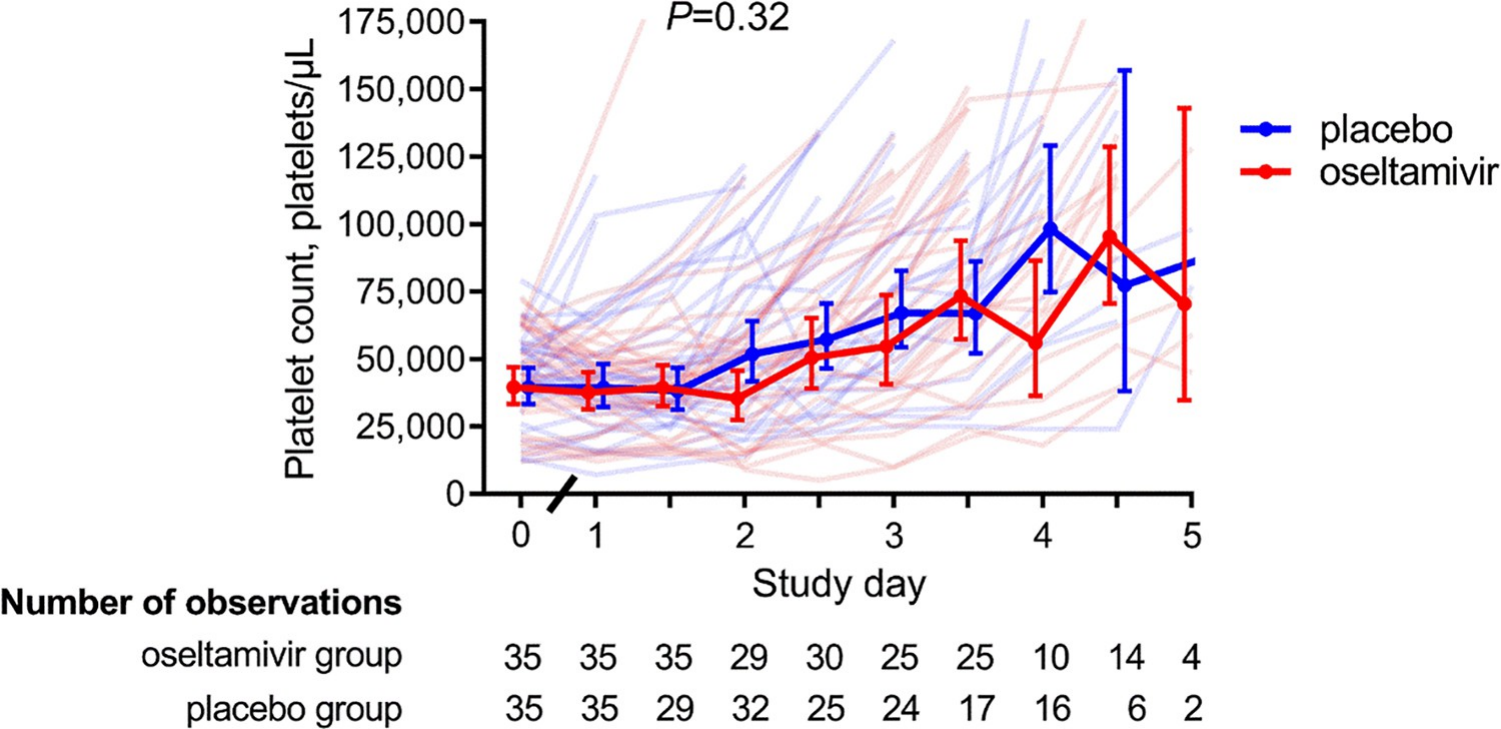
Twice daily platelet count. Data depicted are individual values (transparent lines) and geometric mean (solid line) with 95% confidence interval. *P* value calculated using mixed model method.

The primary combined outcome measure, time to platelet count >100,000/μl or discharge, could be assessed in 64 patients using the intention to treat analysis. Fifty-five patients (27 in the oseltamivir group and 28 in the placebo group) reached a platelet count of 100,000/μl or above. Nine patients (four in the oseltamivir group and five in the placebo group) were discharged with a platelet count below 100,000/μl, of whom three had a platelet count between 95,000 and 99,000/μl. The time to the composite primary endpoint was similar across the groups: 3.0 ± 0.8 days in the oseltamivir group and 2.9 ± 1.1 days in the placebo group (*P* = 0.055). In the 55 patients who reached a platelet count >100,000/μl, there was also no difference in time to reach this time point (oseltamivir group 2.9 ± 0.8 days vs. placebo 2.6 ± 1.0 days; *P* = 0.17). The kinetics of the increase in platelet count between the groups was similar as well (**Fig 2**; *P* = 0.32). The median (IQR) change from baseline platelet count in the placebo group was -3,000/μl (-13,000 to 9,000/μl) at 24 hrs. after start of study medication and 17,000/μl (-7,500 to 44,750/μl) at 48 hrs. In the oseltamivir group, these changes were 0/μl (-9,000 to 3,000/μl; *P* = 0.6 vs. placebo group) at 24 hrs. and -3,000/μl (-16,000 to 15,500/μl; *P* = 0.06) at 48 hrs. The change in platelet count at day five was not calculated because only two and four patients in the placebo and oseltamivir groups were still hospitalized at that day. In participants with a baseline platelet count ≥ 20,000/μl (n = 61), marked thrombocytopenia below 20,000/μl developed in two patients in the placebo group and in six patients in the oseltamivir group. There were no differences in bleeding manifestation (skin petechiae, epistaxis and melena) across both groups during hospitalization (**S1 Table**). Finally, we evaluated whether the time between symptom onset and onset of the intervention might have impacted the results. Adding ‘time since symptom onset’ as a covariate in a linear multivariate model did not result in a significant difference in the primary outcome between the groups (*P* = 0.25).

### Plasma leakage parameters

**Fig 3:**
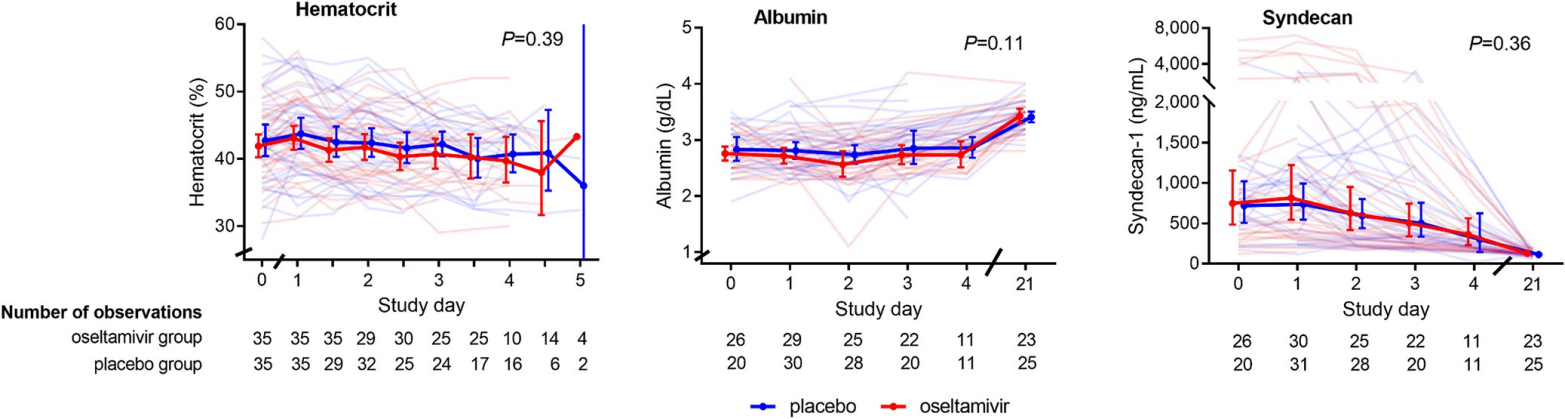
Daily hematocrite, serum albumin and syndecan-1 concetrations. Data depicted are individual values (transparent lines) and geometric mean (solid line) with 95% confidence interval. *P* value calculated using mixed model method.

**Fig 4:**
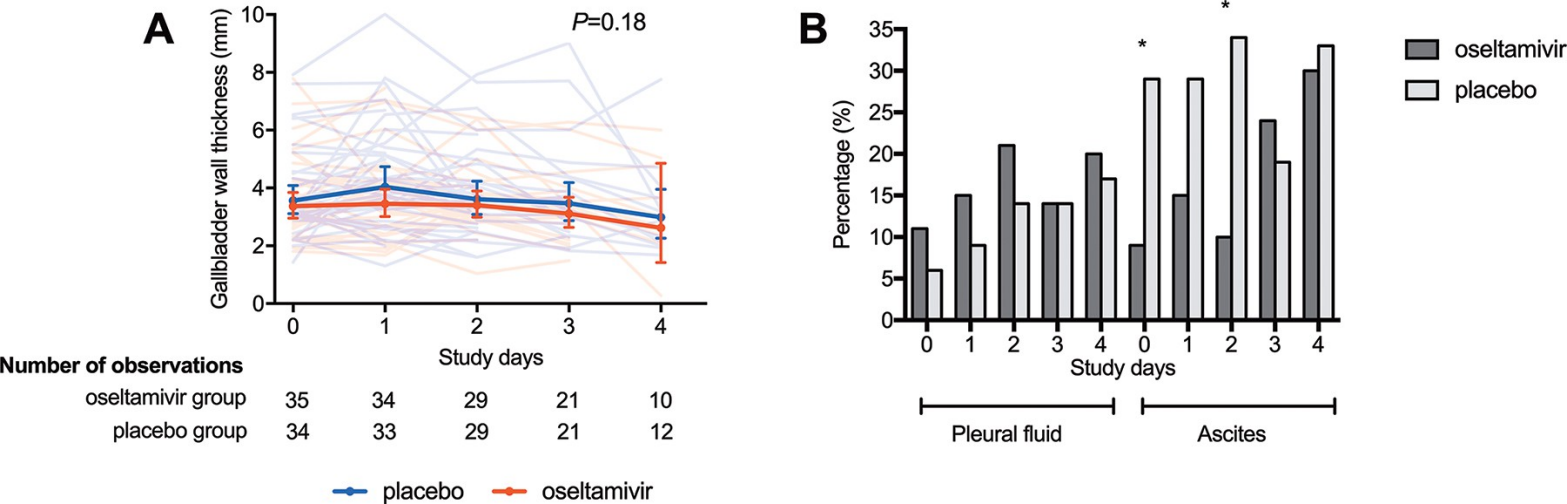
Daily gallbladder wall thickness and presence of pleural fluid or ascites. Gallbladder wall thickness and presence of pleural fluid or ascites was assessed daily by ultrasonography. Gallbladder thickness data are depicted as individual values (transparent lines) and geometric mean (solid line) with 95% confidence interval. *P* value for gallbladder thickness calculated using mixed model method. Differences in presence pleural fluid or ascites calculated using chi-square test (*P<0.05).

Plasma leakage was assessed by a combination of laboratory parameters and daily bedside ultrasonography. The hematocrit and plasma concentrations of albumin and of the glycocalyx marker syndecan-1 were similar between the oseltamivir and placebo groups at the different time points (**Fig 3**). In addition, thickness of the gall bladder wall was similar between the groups (**Fig 4**). At enrollment, pleural fluid was more common in the oseltamivir group, whereas ascites was more common in the placebo. These differences persisted until day 3 of follow up with ascites remaining significantly more common in the placebo group (**Fig 4)**.

### Safety

Treatment with oseltamivir was generally well-tolerated. The incidence of reported side effects in the oseltamivir was similar to the control group (**Table 2**). Mild elevations in serum transaminases were common in both groups. In one event, oseltamivir was interrupted at day 3 due to an ALT concentration more than 10 times the upper limit of normal (614 U/L), which normalized thereafter. One death occurred in the oseltamivir group; this participant was enrolled on the basis of a positive result of dengue IgM with negative NS1 RDT. The patient received two dosages of oseltamivir before this was stopped because of suspected septic shock with progressive multi-organ failure. The participant died on day 3; death was considered unrelated to the study drug.

**Table 2:** Reported side effects.

Side effects, n (%)	Oseltamivir	Placebo	*P* value
**Nausea**	9 (26)	11 (31)	0,8
**Vomiting**	8 (23)	6 (17)	0,8
**Headache**	3 (9)	1 (3)	0,6
**Abdominal pain**	5 (14)	3 (9)	0,7
**Coughing**	1 (3)	0 (0)	0,99
**Diarrhea**	1 (3)	2 (6)	0,99
**Dizziness**	1 (3)	0 (0)	0,99
**Itching**	1 (3)	1 (3)	0,99
**ALT >10x ULN** **ALT >5xULN** **Creatinine >0.5 mg/dl**	1 (3) 2 (6) 2 (6)	0 (0) 3 (9) 2 (6)	0,99 0,8 0,8

ALT, alanine-aminotransferase; ULN, upper limit of normal

## Discussion

In this trial involving adult thrombocytopenic patients, hospitalized with acute DENV infection, adjunctive therapy with oseltamivir phosphate did not shorten platelet recovery time compared with placebo. The trial also did not show an effect of adjunctive oseltamivir on plasma leakage parameters.

Transient thrombocytopenia with hemorrhagic manifestations and plasma leakage are hallmarks of DENV infection. The hypothesis that the sialidase inhibitor oseltamivir may serve as a possible therapeutic adjunct to shorten platelet recovery time and decrease plasma leakage in dengue was based on earlier findings suggesting a role for human sialidases in dengue-associated platelet clearance and endothelial hyperpermeability [16,17].

We previously showed a reduced binding of *Sambucus nigra* lectin (SNA) and *Maackia amurensis* lectin II (MAL-II) to sialic acid residues on the platelet surface of dengue patients relative to patients with non-dengue febrile illness or healthy controls [16]. This loss of sialic acid was caused by translocation of platelet neuraminidase-1 (Neu-1) to the platelet membrane following binding of plasma von Willebrand factor to platelet glycoprotein (GP)-1b. *In vitro*, oseltamivir was shown to lower sialidase activity and β-galactose exposure (indicative of reduces desialylation) at the platelet surface [16,27]. This was further confirmed in a patient with anti-GP1b antibody-mediated ITP, in whom oseltamivir phosphate reduced desialylation of platelet glycoproteins [23]. Different retrospective studies in conditions with accelerated platelet clearance, such as anti-GPIb antibody-mediated ITP [22,23] or suspected influenza [24], and an open-label randomized trial in sepsis [26], have supported the notion that Neu-1 inhibition by oseltamivir increases platelet counts. This was further supported by the results of a recent multicentre, randomized, open-label phase 2 trial, which showed that oseltamivir in combination with dexamethasone resulted in a better platelet response compared with dexamethasone alone in patients with ITP [25]. Besides platelet neuraminidase, DENV NS1 has been shown to directly activate endothelial neuraminidases leading to disruption of endothelial glycocalyx components [17]. The neuraminidase inhibitor zanamivir partially prevented DENV NS1-induced endothelial hyperpermeability in endothelial cell monolayers [28]. Syndecan-1 is a component of the endothelial glycocalyx and the strong increase in plasma syndecan-1 concentrations in the participants in our study reinforces the importance of glycocalyx disruption in DENV infection.

The reasons that oseltamivir had no apparent effect on platelet counts, markers of plasma leakage and glycocalyx distortion in this study remain speculative, but may involve one or more of the following; first, dengue-associated thrombocytopenia and plasma leakage are both multifactorial in origin and targeting neuraminidase alone may be insufficient to impact these processes. Second, oseltamivir phosphate was designed to inhibit viral neuraminidase, and data of its inhibitory actions on human neuraminidases are inconclusive. In fact, whereas different studies have provided evidence of oseltamivir inhibiting mammalian neuraminidases [23,29], Hata *et*. *al*. did not observe a substantial inhibiting effect of oseltamivir on recombinant soluble human neuraminidases [30]. Whether other neuraminidase inhibitors, such as zanamivir, would have yielded different results remains speculative. Third, our study did not include the pharmacokinetics of oseltamivir phosphate. Data from preclinical studies regarding the required dose of oseltamivir to exert the desired platelet effects is limited. However, the fact that a standard dose of 75mg BID was sufficient in different observational and interventional studies to exert an effect on platelet number, as well as to reduce platelet desialylation (assessed by flow cytometry) in a chronic ITP patient, suggests that the standard oseltamivir phosphate dose is sufficiently high [20–27]. Fourth, we cannot exclude that oseltamivir prevents or limits the development of thrombocytopenia when given earlier in the course of DENV infection, although we consider this scenario unlikely given the complete absence of difference in platelet counts between the two groups in this study.

Limitations of our study include the fact that assessment of changes in sialic acid residues on the platelet membrane was not feasible in the trial participants. This assessment requires a rather complicated flow cytometry assay that needs to be performed on fresh blood samples within hours after blood sampling. With six participating centers, this was logistically not feasible, but it should be considered in future studies as it may yield important mechanistic information. In addition, molecular confirmation of DENV infection with serotype identification was not performed in our study.

In conclusion, in adult patients with DENV infection and thrombocytopenia (<70,000/μl), adjunctive therapy with oseltamivir phosphate did neither improve platelet recovery, nor affect plasma leakage parameters.

## Supporting information

S1 Fig Number of study medication doses taken by every participant. (DOCX)

S1 Table Bleeding complications during admission. (DOCX)
