# Supplementary material for: Effect of oseltamivir phosphate versus placebo on platelet recovery and plasma leakage in adults with dengue and thrombocytopenia; a phase 2, multicenter, double-blind, randomized trial
Source: PLoS Negl Trop Dis. 2022 Jan 7;16(1):e0010051. doi: 10.1371/journal.pntd.0010051 (PMC8789129; doi:10.1371/journal.pntd.0010051)
Supplement: S1 Fig — (DOCX) [file pntd.0010051.s001.docx]

**S1 Fig.** Number of study medication doses taken by every participant.
