# Supplementary material for: Effect of oseltamivir phosphate versus placebo on platelet recovery and plasma leakage in adults with dengue and thrombocytopenia; a phase 2, multicenter, double-blind, randomized trial
Source: PLoS Negl Trop Dis. 2022 Jan 7;16(1):e0010051. doi: 10.1371/journal.pntd.0010051 (PMC8789129; doi:10.1371/journal.pntd.0010051)
Supplement: S1 Table — (DOCX) [file pntd.0010051.s002.docx]

**S1 Table.** Bleeding complications during admission.

|  | Bleeding | Oseltamivir | Placebo |
| --- | --- | --- | --- |
| Day 0 | Epistaxis | 5 | 3 |
|  | Skin petechiae | 10 | 12 |
|  | Melena | 1 | 1 |
|  | Hematuria | 2 | 0 |
| Day 1 | Epistaxis | 4 | 1 |
|  | Skin petechiae | 6 | 7 |
|  | Melena | 0 | 2 |
|  | Hematuria | 3 | 0 |
| Day 2 | Epistaxis | 2 | 0 |
|  | Skin petechiae | 3 | 4 |
|  | Melena | 0 | 0 |
|  | Hematuria | 1 | 0 |
| Day 3 | Epistaxis | 0 | 0 |
|  | Skin petechiae | 4 | 4 |
|  | Melena | 0 | 0 |
|  | Hematuria | 0 | 0 |
| Day 4 | Epistaxis | 0 | 0 |
|  | Skin petechiae | 1 | 0 |
|  | Melena | 0 | 0 |
|  | Hematuria | 0 | 0 |

Differences between groups were statistically not significant.
